# Archaeological evidence of resource utilisation of the great whales over the past two millennia: A systematic review protocol

**DOI:** 10.1371/journal.pone.0295604

**Published:** 2023-12-14

**Authors:** Danielle L. Buss, Youri van den Hurk, Mohsen Falahati-Anbaran, Deirdre Elliott, Sally Evans, Brenna A. Frasier, Jacqueline A. Mulville, Lisa K. Rankin, Heidrun Stebergløkken, Peter Whitridge, James H. Barrett

**Affiliations:** 1 Department of Archaeology and Cultural History, NTNU University Museum, Trondheim, Norway; 2 Department of Archaeology, Memorial University of Newfoundland and Labrador, St John’s, Newfoundland, Canada; 3 MSDS Marine and MSDS Heritage, Holbrook, United Kingdom; 4 Nova Scotia Museum, Halifax, Nova Scotia, Canada; 5 School of History, Archaeology and Religion, Cardiff University, Cardiff, United Kingdom; 6 Department of Historical and Classical Studies, NTNU, Trondheim, Norway; New York State Museum, UNITED STATES

## Abstract

Archaeological faunal remains provide key insights into human societies in the past, alongside information on previous resource utilisation and exploitation of wildlife populations. The great whales (Mysticete and sperm whales) were hunted unsustainably throughout the 16th - 20th centuries (herein defined as the modern period) leading to large population declines and variable recovery patterns among species. Humans have utilised whales as a resource through carcass scavenging for millennia; however, increasing local and regional ethnographic and archaeological evidence suggests that, prior to the modern period, hunting of the great whales was more common than previously thought; impacts of earlier hunting pressures on the population ecology of many whale species remains relatively unknown. Hunting guided by traditional ecological knowledge may have been sustainable and likely originated in societies that also incorporated opportunistic use of stranded individuals. The collation of georeferenced zooarchaeological data of the great whales between the 1st - 20th centuries CE worldwide will provide insight into the timescale and distribution of resource utilisation of the great whales and how this varied within and between societies, and may have changed over time. By comparing regions of known resource utilisation and breeding and feeding grounds of current-day whale populations, this information will subsequently be used to infer regions where whale populations were possibly lost or extirpated prior to detailed historical records. This systematic review protocol also provides a template for archaeologists, ecologists, and historians interested in using faunal remains to infer historical ecology and resource use of wild animal populations. The transparency of our data collection approach provides opportunities for reproducibility and comparability with future datasets.

## 1. Background

Colonisation of uninhabited land masses by humans over time, alongside major changes in resource exploitation (e.g. the transition from hunter-gatherer to agricultural societies) [1–3], is often associated with the depletion of terrestrial megafauna worldwide [4–7]. This is unsurprising given human colonisation likely resulted in increased hunting pressures, land-use changes resulting in habitat destruction (e.g. clearing of forest for agriculture) and the introduction of non-native species (e.g., rats/cats) [8–11]. Although it is often challenging to link causality between human arrival and species extinctions, there is substantial correlative evidence that colonisation of European settlers directly and/or indirectly impacted wild-animal populations across the globe [8], although we note here that there are a few case studies where this correlative association is not well supported (e.g., [12,13]).

Between the 19th century and the present, the oceans underwent a similar transition; marine resources were exploited at unsustainable rates [14,15] and the expansion of large-scale cultivation of aquatic resources (e.g. fish farming/aquaculture) continues even now [16–19].

Prior to and during this globalization era, major socio-economic transitions resulted in changes in the use of marine resources as commodities [20–25]. For example, subsistence hunts and/or local fisheries expanded towards large-scale commercial fisheries and international trade [22,24,26–28], whilst local subsistence use of marine mammals expanded into a global market for fur pelts, partially driven by demand from the fashion industry [29–35].

Increased use of marine commodities can either result in sustainable subsistence practices or lead to the depletion of wild animal populations [36–41]. For example, there is evidence of pre-Inuit groups in North America likely utilising bowhead whales for millennia (e.g., [42]). Inuit expansion across the Arctic around approximately 1200 CE was concurrent with the expansion of specialist bowhead whale hunting [43–46]. This resulted in a sustainable whale fishery that continues in parts of the Arctic to the present day [47,48]. In contrast, the global trade of walrus material from Greenland transitioned from sustainable subsistence harvesting in the eleventh century to overexploitation by the thirteenth century, following increased demand for walrus ivory across Eurasia [49,50]. Information regarding past exploitation patterns thus informs our understanding of human-wildlife interactions and how sustainable subsistence hunting and/or overexploitation came to be.

The accelerated use of animal populations can sometimes follow a pattern of serial depletion [51,52]. Serial depletion is the sequential reduction in abundance of exploited taxa, often entailing the use of more and more distant populations. Indeed, serial depletion of Svalbard’s marine mammals (bowhead whales, *Balaena mysticetus*, walrus, *Odobenus rosmarus*, and polar bears, *Ursus maritimus*) has been documented following the arrival of English and Dutch whalers in the 17th century, alongside Norwegian hunters and Russian Pomors by the early 18th century [53–55]. Further research is required to properly understand the impact of ecological globalization on sea mammal populations and identify any evidence of historic serial depletion events worldwide.

Resources provided by the great whales included oil, meat, and structural materials (bone, baleen) used for fashion, upholstery and building infrastructure (e.g. [44,56–58]). Large volumes of oil were produced by boiling vast quantities of blubber, predominantly used for heating, lighting, margarine and transmission fluid, whilst a variety of tissues (e.g., muscles) were consumed as meat from fresh carcasses [59–65]. Archaeological and ethnographic evidence suggests that the great whales (baleen whales, Mysticeti and the sperm whale *Physeter macrocephalus*) have been a global resource for human societies, either accessed through hunting or via carcass scavenging, for millennia [42,43,47,48,66–73]. The earliest written accounts of whaling include 670 CE in Continental Europe, 731 CE in England, and the late 9^th^ century in Scandinavia ([74] and refs within); however, petroglyphs of humans capturing or standing over whales with spears date back to the Neolithic in some regions, and have been dated alongside harpoons and whale bones [72], indicative of active hunting of whales since approximately 7000 years before the present. The long-term importance and association of specific species to coastal indigenous communities is also evident from archaeological and historical records. For example, bowhead whales were targeted by indigenous communities in Chukotka (Russia) and St Lawrence Island (Alaska) since at least the 6th century CE and by Inuit communities by the 12th century CE [75]. Similarly, European societies targeted cetaceans in local waters by the 7th century CE [65], with whaling widespread in Basque, Norman and Flemish societies predominantly in European waters by the 11th century CE [76–80]. By the 16th century, European whalers were exploiting whale stocks in the western North Atlantic [63,81–83]. It has been suggested that cross-oceanic expansion of whale fisheries by the 16th century was potentially driven by resource depression of whales in the eastern North Atlantic prior to this period. This hypothesis is supported by the local extirpation of the North Atlantic right whale, *Eubalaena glacialis*, in the eastern North Atlantic [84]; the population size of *E*.*glacialis* in the western North Atlantic now remains at a few hundred individuals with low levels of genetic diversity evident [85–87] and the threat of extinction [88,89]. Whaling expanded globally between the 16th - 20th centuries, with whaling from the 18th century onwards resulting in population declines and serial depletion of whale species worldwide [41,67,78,90–92]. The contribution of human societies to declines in whale populations prior to the well-documented ‘industrial’ whaling era (18th century onwards) are still being discovered (e.g. [70,93–95]). For example, projects are underway to further understand the interaction between human societies and the demise of the gray whale, *Eschrichtius robustus*, which went extinct from the North Atlantic most likely by the 18th century [84,95,96].

Despite evidence of whales being utilised for resources (via live hunting or opportunistic scavenging of strandings) for millennia [60,61,64,65,80,91,95,97–99], the ecological and societal implications of pre-industrial whaling has only been documented on a localised scale (e.g. [70,73,80,99–102]). The majority of the great whales (baleen and sperm whales) undertake vast annual migrations (often between higher latitude feeding areas and lower latitude wintering/breeding areas), with the possibility of being hunted or scavenged by multiple past human societies along their migratory range [59,103,104]. Therefore, localised studies have limited capacity to infer the relationship between human societies and specific whale species and populations (i.e. across the full migratory range). Evidence of whale resource use by humans at a broader scale may help to inform when and where past whaling or scavenging events could have occurred.

Rates of change in utilisation quantities can be inferred from the number of identified bone specimens (NISP) known from archaeological sites. NISP can be used to infer acceleration of marine resource utilisation over time and for instances when hunting is known to have occurred, rates of past extractions can be estimated [105–107]. Indeed, this approach has been used previously to document the onset and acceleration of marine fishing and trade in medieval Europe [22,108], and to document the exploitation and extirpation of several avian taxa, including the New Zealand moa (Dinornithiformes) [109], and the flightless duck, *Chendytes lawi* [110]; these are just two examples among many others that exist (e.g., [111–114]).

Zooarchaeological NISP data of whales over large spatiotemporal scales will help to inform how human societies utilised the great whales for resources and may have impacted great whale populations across their distributions and migratory ranges and/or during transitions from local subsistence to global commodification. However, it is challenging, and in some instances impossible to distinguish between archaeological bones of stranded versus hunted individuals. Strandings of large whales is infrequent with the majority of reported mass strandings related to smaller odontocete species (e.g., [115–119]). Variation in life-history and social structures of cetaceans is one likely driver of the relatively higher rates of strandings in smaller cetacea species (see [115,120]). In contrast, life history strategies of most large baleen whale species are biased towards off-shore foraging and migration through the open-ocean [59,121], where they are more likely to die at sea providing essential nutrients to deep-sea species as whale falls [122,123]; although large baleen whales are known to strand on occasion [115–119] and are therefore likely to be represented in the archaeological record. Previous research has shown that in some instances it is possible to differentiate between whale assemblages corresponding to natural stranded whales versus hunted whales, with the reporting of whale hunting equipment now reported as evidence [124]. Despite the challenges of separating archaeological bones from stranded versus hunted individuals, spatiotemporal variation of whale faunal remains will provide the opportunity to detect ecological responses (changes in NISP of the great whales) to past climatic regimes.

Archaeological NISP data can also be used to identify specific areas of intensified resource utilisation (herein referred to as ‘hotspots’ following [125]) alongside relative changes in intensification (e.g. [111,126]). The latter has previously been referred to as an ‘event horizon’, whereby a clear and often dramatic shift in resource use is evident within the zooarchaeological record [127]. Indeed, the ‘fish event horizon’ is a term used to describe a shift from the harvesting of predominantly freshwater to marine fish initially attributed to the 10th and 11th centuries [22], a term which has subsequently been adopted by the archaeological community (e.g. [128–131]). Marine event horizons have been defined as rapid onsets of widespread marine consumption [96], and they have been viewed as an innovation phase that often preceded further accelerations of marine extractions (e.g. the emergence of early modern commercial fisheries in Newfoundland). Relative changes in the quantities of archaeological whale material may be able to identify when and where event horizons, accelerated extractions and hotspots of whale resource use have occurred in the past. These data in combination with other evidence, such as historical documentation of whaling events and/or practices might provide insight into the transition from local subsistence to global commodification, alongside the opportunity to detect ecological responses to climatic change.

### 1.1 Objective

The primary objective of this systematic review is to identify zooarchaeological evidence of the great whales between 1 CE and 1900 CE (the start of the 20th century whaling period) and use this information to identify spatiotemporal variation in the occurrence and acceleration of the resource use of whale products worldwide. The time periods between 1 CE and the present day were characterised by large-scale environmental, demographic and societal change, including (but not exclusive to), the rise and expansion of Eurasian empires [132], expansion of Arctic indigenous communities [43,46,75,133], societal shifts in economic practices and resource procurement worldwide (e.g., [134–137]), environmental fluctuations including, the Roman Warm Period, the late Antique Little Ice Age, the Medieval Climate Anomaly, the Little Ice Age, and the start of the industrial revolution [138–144]. Although whales were also an important global commodity from 1900 CE, whaling and whale resource use has been well-documented during this time [59–61,91,92,97,145] and therefore, we will not re-document this evidence.

We will use zooarchaeological evidence to address four main research questions:

**1.** To what taxonomic level are marine mammal faunal remains recorded and how many of these are likely to correspond with the great whales (collectively: Mysticeti and *Physeter macrocephalus*)?
**2.** To record when and where there is evidence of resource utilisation of the great whales between 1 CE and 1900 CE across the globe.
**3.** To identify event horizons, accelerated extractions and spatiotemporal hotspots of whale resource use worldwide between 1 CE and 1900 CE.
**4.** To identify environmental and climatic correlates of regional whale resource use worldwide and where possible to evaluate to what degree the observed patterns were associated with concurrent socio-economic change.

In combining the research traditions of zooarchaeological synthesis [146–148] with formal systematic review methodology (e.g. [149–151]), we hope this transparent protocol will also provide a road map for researchers aiming to use zooarchaeological data to infer the past resource utilisation of other marine taxa over broad spatiotemporal scales.

## 2 Methods

Here we describe the data sources, search strategy, study inclusion criteria, quality control assessments and then go on to discuss some of the common biases associated with zooarchaeological count data.

### 2.1 Data type

During archaeological research, faunal remains are typically collected, taxonomically/anatomically identified, and the number of identified specimens (NISP) recorded. This is usually in the form of preserved (often fragmentary) bones and/or teeth of fauna, although proteinaceous tissues such as keratin (thus baleen for the Mysticeti), alongside skin and hair are also sometimes preserved under special conditions (e.g. under anoxic or cold conditions—permafrost). Artefacts or architectural elements composed of faunal material are also collected but often reported separately. In this review, bones, teeth (*Physeter macrocephalus* only) and baleen (Mysticeti only) will be recorded in the form of NISP from the archaeological literature; where reported, the number of architectural whale bones and of artefacts worked from whale material will also be recorded. NISP is one of the two most common proxies for specimen abundance in zooarchaeology and is more consistently reported globally than others (e.g. minimum number of individuals, minimum number of elements, minimum animal units) [148,152–154]. It is accepted that NISP is an imperfect proxy for the historic abundance of fauna; it is key to note that NISP is subject to numerous biases, including, but not exclusively, problems associated with differential fragmentation and transportation [148]. In instances where NISP counts of artefacts or ecofacts are unreported, presence or absence will be recorded.

### 2.2 Search strategy

Scoping searches using Web of Science (WoS), Scopus and Google Scholar conducted in July 2022 indicated that there are numerous published reports available online that contain NISP data on faunal remains of whales at archaeological sites on a broad spatiotemporal scale. We will systematically search these databases for relevant sources from the published literature using the search terms below. Additionally, to identify relevant literature written in non-English, translated versions of these search terms will be included (see S1 Table for a full list of non-English search terms). To identify relevant publications that may not be included in the indexes of WoS, Scopus and Google Scholar, these search terms will also be included in searches of researchgate.net, jstor.org and academia.edu (websites where academics house their existing research portfolios). For each identified study, the title, abstract (or equivalent), and where relevant, key words, will be scanned by a post-doctoral researcher to assess whether the study is likely to contain information on faunal remains of the great whales over the past 2000 years. Studies that meet the inclusion criteria (see section 2.3 below), will be searched to identify further literature using forward and backward chasing (upward citations) using the R Package CitationChaser [155] and using traditional searches by eye [156,157]. Although unpublished reports include much existing information in archaeology, they are variably accessible, sometimes ambiguous in terms of intellectual property status and the quality of data difficult to determine, given that they have not undergone the peer-review process [158,159]. Therefore, data from unpublished reports identified from upward and backward chasing, or from academic portfolio web page searches, will not be included.

#### Search terms

(Archaeology OR Archaeological OR Archeology OR Archeological) AND (Number of identified specimens OR NISP OR numbers OR nrs OR Fauna OR Zooarchaeology OR Archaeofauna OR Archaeozoology) AND (whale OR baleen OR whalebone OR cetacea OR Mysticeti OR rorqual OR Balaenidae OR Balaenoptera OR Megaptera OR Physeter).

To decide which languages to include in international search terms, the words ‘archaeology/artefact/history’ and ‘whale’ were translated into the top 30 globally most spoken languages (available online: https://www.visualcapitalist.com/100-most-spoken-languages/, accessed on 5 January 2023) and the number of hits that appeared in Google Scholar recorded. Search terms were retained for languages with over 1000 hits in Google Scholar. Additionally, languages of nations that are still known to hunt whales and were not in the world’s list of top 30 most spoken languages were also included (Danish, Faroese, Greenlandic, Norwegian, Icelandic, Inuktitut and Iñupiaq). A full list of non-English search terms is shown in S1 Table.

### 2.3 Study inclusion criteria

Studies will only be included if they meet the following criteria:

1. Quantitative data are available from archaeological sites in the form of the number of identified specimens (NISP; as originally defined by the zooarchaeologist(s)) relating to either ‘large unidentified whale’ or taxa within one of the following taxonomic groups: Mysticete or Mysticeti (baleen whales); Physeteridae (sperm whales).
2. Chronological information is available for the relevant whale specimens, or can be inferred from the associated site where they were originally identified, and they date between 1 CE and 1900 CE, with reported or inferred chronological precision of ≤500 years. When date ranges of ≤500 years span BCE and CE, specimens will be included if the chronological midpoint postdates 1 CE.
3. Geolocations for each site are reported or can be identified from reported site information within a minimum precision of 1.0 decimal degrees.

### 2.4 Data extraction and management

**Fig 1:**
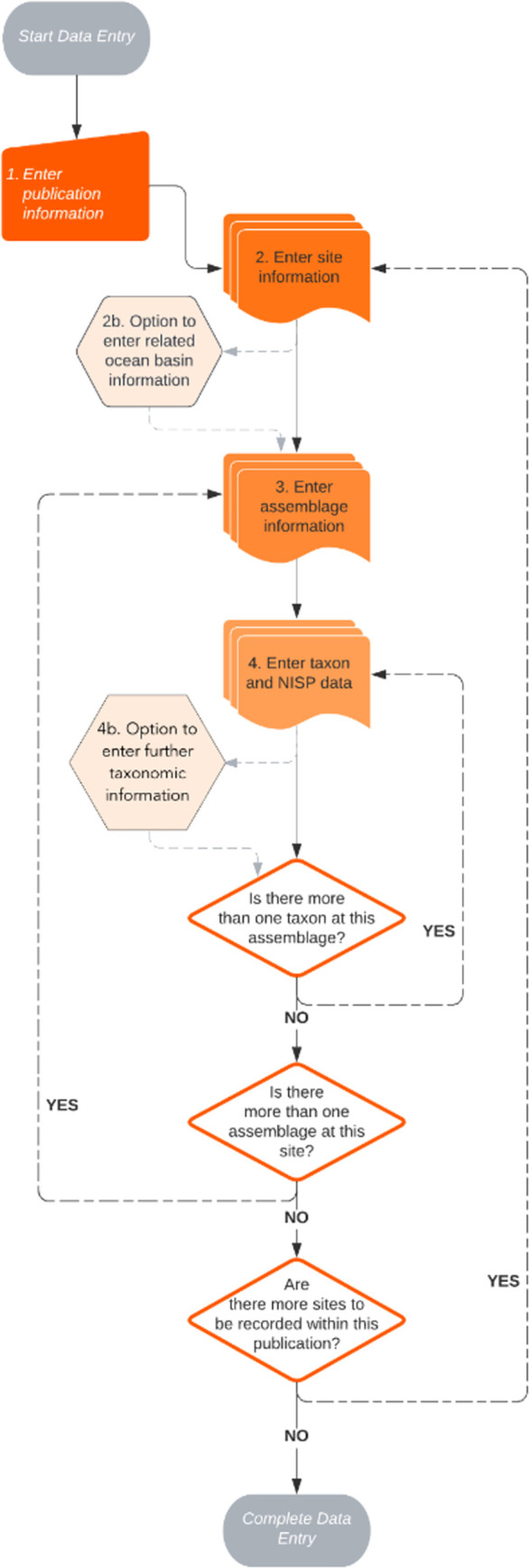
Schematic of data entry of NISP and associated metadata into the whale bone database.

All reports that meet eligibility criteria will be downloaded, or the relevant hard-copy sections (if available) scanned to PDF, within the limits of copyright. All eligible and accessible references with associated PDFs will be imported to the referencing manager and duplicates removed. Data will be manually added from individual reports into a custom-designed OpenOffice 4.1.13 database [160] following the protocol shown in Fig 1. The data fields to be extracted from each report are shown in S2 Table (essential) and S3 Table (non-essential). In summary, data will be extracted on the following:

#### Publication data

Publication year, title of publication (journal, book etc.), title of section (subchapter, chapter or article), publisher and place for books, authors, corresponding author name and email, DOI (where available), URL if no DOI exists (where available), type of publication and intellectual property status, name of the researcher that performed faunal analysis, and the source of alternative report(s) if chronology or other metadata (e.g. georeference) are reported elsewhere.

#### Site location(s) and chronology

Site location(s), decimal latitude and longitude coordinates, country, province/state/or equivalent (e.g. county), site type (e.g. shipwreck, refuse pit), archaeological site name, archaeological site number or code (where applicable), modern settlement name (where applicable), assemblage name(s), assemblage date(s) (verbatim as reported, often using regional archaeological terminology), assemblage start and end dates (CE—CE), the method(s) used to determine assemblage/site chronology, the method used to recover faunal material (optional, if reported, but not necessary for large cetacean remains).

#### Taxon and NISP data

Taxon as reported (e.g. large unidentified whale), number of identified specimens (NISP) per taxon, the total number of identified specimens in the assemblage (where applicable), the total number of unidentified specimens in the assemblage (where applicable) and which taxonomic groups this includes (e.g. all vertebrates or only mammals), an indication of whether other taxonomic groups (of the following: birds; fish; terrestrial mammals) were reported within the same study. The total number of identified mammal specimens in the assemblage will be recorded in additional to the totals of larger taxon groups (e.g., all vertebrates) (where applicable).

#### Evidence of whaling equipment

Where available, the presence or absence of whale hunting equipment at an archaeological assemblage will be recorded, alongside a description of the equipment (e.g. large rendering ovens).

### 2.5 Potential biases and quality assessments

Variable butchery practices, transport practices, preservation conditions, excavation methods, dating methods and research intensities are all known to impact the precision and accuracy of zooarchaeological data recovery; all are often challenging to assess post-study, simply due to a lack of reporting on these topics [161,162]. For example, the experience, methods, and reference materials available to individual faunal analysts can greatly impact both the precision and accuracy of zooarchaeological identifications (e.g. [163–166]). Moreover, the likelihood of whether an animal specimen has been traded and therefore ended up much further afield from the site of harvest should be considered. For example, in the 18th century, baleen was routinely transported from whale hunting locations in far northern Labrador to trading centres in central and southern Labrador [167,168], and Inuit groups in the Mackenzie Delta in the Northwest Territories engaged in inter-regional transport and trade of prepared whale products from the outer to the inner coast and to the interior [169]. Where practicable, potential ambiguities regarding chronology, taxonomy and site location will be evaluated using quality criteria on an ordinal scale (1–3; weak, medium, strong; 0—data recorded but will not typically be used in analysis). Details of this data quality scoring are outlined below in sections 2.5.1 to 2.5.3.

#### 2.5.1 Dating

Chronological information will be recorded as reported for the faunal element(s), or as reported for the assemblage from which the faunal element was obtained. Chronological control of excavated whale specimens is occasionally available from direct archaeometric dating, however, radiocarbon calibration is problematic for migratory marine taxa that often frequent high latitudes because of variable air-sea gas exchange due to differing wind strength and sea ice cover, alongside ocean currents resulting in different ‘aged’ surface waters [170,171]. Thus, the chronology reported is frequently derived by combining information from stratigraphy, associated artefacts, archaeometric dates on associated terrestrial materials and in some instances ancillary information, such as the presence of raised shorelines dated based on sea level curves (e.g. [172,173]). When radiocarbon dating is used, researchers may adjust for the ‘marine reservoir effect’ using locally derived corrections known as ΔR in association with a global marine calibration curve (e.g. the Marine20 [171]). However, as the majority of the great whales are highly migratory and often forage across large latitudinal and longitudinal ranges, with variation in foraging strategies differing between individuals and even within individuals between seasons, it is challenging to precisely correct ^14^C dates obtained from whale bones. As a result, the precision from the archaeological dating methodology is often vague and the true error range not always known. In this review, dates will be accepted as reported in the published literature, with reliability scored based on the clarity, methodology and types of archaeological evidence employed. The quality of the chronological of information will be assessed using two categories, chronological timespan, and methodology (see below).

#### A. Chronological timespan

1. Assemblages with a chronological range of >500 years will be classified as temporally uninformative (0).
2. Assemblages with a chronological range of 301–500 years will be classified as data quality (1).
3. Sites with a chronological range of 201–300 years will be classified as data quality (2).
4. Sites with a chronological range of < 300 years will be classified as data quality (3).

#### B. Chronological methodology

Faunal remains are often associated with chronological information gathered from contexts using archaeometric and other accurate and replicable (albeit not always precise) methods, such as dendrochronology, radiocarbon dating, stratigraphy, and numismatic/typological dating, making it possible to infer likely time periods.

1. Assemblages with no reported date will be classified as temporally uninformative (0).
2. Assemblages with an estimated date, but when the dating method used is not clearly reported, will be classified as data quality (1).
3. Sites (and associated assemblages) that were dated using typology, stratigraphy and/or chronometric methods, but without quantified and up-to-date estimates of error, will be classified as data quality (2).
4. Sites using chronometric methods that report primary data (e.g. radiocarbon assays that can be recalibrated) will be classified as data quality (3).Where uncalibrated radiocarbon dates are available they will be recalibrated following current best practice in downstream analysis.

#### 2.5.2. Taxonomic identification

The identification of whale bone specimens to a high taxonomic level (e.g. species/genus) from archaeological contexts is challenging due to the limited research that has been conducted using osteological morphological criteria to separate whale species, the rarity of extensive osteological reference collections, and the frequently fragmented state of whale bones. This has often led to whale bone specimens merely being identified as “large unidentified whale” or “unidentified cetacean” (e.g. [174]); for an extensive discussion on the challenges associated with taxonomic identification of animal skeletal remains see [165]. Previous research utilising molecular techniques have indicated that whale bone specimens taxonomically identified using traditional zooarchaeological approaches have sometimes been taxonomically misidentified. For example, peptide finger-printing (ZooMS) and ancient DNA analysis revealed that five specimens previously reported as grey whale in fact represented three fin whales, one sperm whale, and one Mysticete [175]. Furthermore, DNA analysis of 16^th^ century whale specimens previously thought to represent North Atlantic right whales were identified as bowhead whales amongst a Basque whaling assemblage in the North Atlantic [67,176]. These results highlight the necessity of validating osteological morphology-based identification practices using molecular methods. Because of the likelihood that there will be random error in taxonomic identifications of whale remains, quality criteria will be assigned as follows:

1. Faunal identifications classified as ‘Unidentified marine mammal or cetacean’ will be classified as taxonomically uninformative (0).
2. Faunal identifications classified as ‘Unidentified large whale’ or equivalent will be classified as data quality (1).
3. Faunal identifications of greater taxonomic resolution (e.g. Mysticeti, Balaenidae, Physeteridae, *Megaptera novaeangliae*) based on zooarchaeological assessments alone will be classified as data quality (2).
4. Faunal identifications to a scientific taxon made using ZooMS, aDNA or specific morphological criteria will be classified as data quality (3).

#### 2.5.3. Location

The precise locations of archaeological assemblages, sites, or features are not always given due to common legalities and when they are legally provided are not always straightforward to interpret. Locations of excavation sites are often reported using a geographic map indicating the location of the excavated site, or a description of the site location is reported in the text. In some instances, the archaeological site name or code is reported which may be associated with a georeference in other sources. Geographical coordinates are often not reported alongside these lines of information. However, Google Earth, a platform that provides open-source high resolution images of the majority of the earth’s surface, can often be compared with reported maps and used to pinpoint the excavated site clearly. However, the resolution of maps for some sites, particularly in non-residential areas are low, making it difficult to obtain precise coordinates. For studies that include only a low-resolution site map in the public domain, where feasible, other (sometimes unpublished) reports will be evaluated to assess the exact location of the site with higher precision, unless this information is noted as confidential. Due to these considerations, the raw zooarchaeological NISP data will be associated with spatial information of varying degrees of precision.

1. Faunal remains without location data (e.g. museum specimens with unknown provenance) will be classified as spatially uninformative (0).
2. Faunal remains associated with a broad spatial scale (e.g. country, province, state) will be classified as data quality (1).
3. Faunal remains lacking a site-specific georeference, but that can be located to within 1 degree of latitude and longitude (for example, attributed to a known modern settlement without specific site coordinates), or where specific site coordinates are provided but of a resolution of 1 degree latitude and longitude, will be classified as data quality (2).
4. Faunal remains associated with specific site coordinates (or described location that can be used to derive such coordinates) with precision exceeding 1.0 degree latitude and longitude will be classified as data quality (3).

## 3. Analytical approach

In centuries poorly served by quantitative records of resource extraction, zooarchaeological records coupled with palaeoclimatic data and a good understanding of local human societies can be used to identify past changes in patterns of animal resource use (e.g. [177]). Here, records of whale bone, teeth (sperm whales only) and baleen dated between 1 CE—1900 CE with known provenance and chronology will be used alongside environmental and cultural correlates to identify spatiotemporal variation in resource use and possible extraction rates of the great whales. These data will be used to determine: (i) at what taxonomic level marine mammal faunal remains are recorded; (ii) when and where there is evidence of human societies utilising the great whales as resources, including the identification of spatiotemporal hotspots, event horizons and acceleration events worldwide between 1 CE—1900 CE; and (iii) environmental and climatic correlates of historic whale resource use. In doing so, zooarchaeological evidence will be treated as a proxy for whale utilisation, although with associated biases, including the transport of large whale bones from locations where carcasses were actively harvested to settlement sites [178–180]. The anticipated analytical approaches are outlined in the subsections below; however, these may be adjusted downstream based on the quantity and quality of the extracted data and any new statistical methods.

### 3.1 The taxonomic level of whale identifications

To investigate differences in the level of taxonomic identifications between time periods and regions, generalized linear models will be carried out with the taxonomic level of identification as the response variable (i.e. species, genus, family, order or pseudo-order e.g. (‘unid. large whale’)). The start date, end date and total date range of the associated assemblage, alongside the methodology used for taxonomic identification (e.g. genetic, peptidefingerprint, morphology) and the location of identification (country/ocean basin) will be included as independent variables. Taxonomy will be standardized across the extracted datasets using the R packages Taxize and rGBIF [181,182] and nomenclature will follow that reported on the Global Biodiversity Information Facility (GBIF).

### 3.2 Temporal and spatial variation in whale faunal remains

To investigate spatiotemporal variation in resource utilisation of the great whales by human societies worldwide between 1–1900 CE, NISP data will be aggregated within hexbins (representative of 1 degree × 1 degree latitude and longitude) and mapped as total counts and as counts per quarter (< 400 CE; 401–900 CE; 901–1400 CE; 1400–1900 CE). When feasible varying degrees of higher chronological resolution will also be used. Spatial aggregations will be implemented using the R packages ggplot2, rgadal and rgeos. From pilot exploration of the available literature, it is highly likely that the taxonomic level of faunal assemblages will vary between regions and centuries. Furthermore, many reports record NISP data as “unidentified large whale” or “baleen whale”, whilst those reported at a greater taxonomic resolution using zooarchaeological assessment (e.g. Balaenidae) are subject to inaccurate identification when not based on more reliable methods (e.g. peptide fingerprinting or genetics) [173]. Thus, all records at more precise taxonomic levels will be collated alongside records of ‘large unidentified whale’ and data analysis also performed at this less precise taxonomic level. Moreover, to investigate temporal variation in global harvests of ‘the great whales’ by human societies worldwide between 1–1900 CE, aoristic sum analysis will be used following [129], using 100 year discrete time bins and the associated R Package archSeries. This approach handles datasets with varied temporal resolution by summing the probability of an event (in this case NISP occurrence) uniformly over discrete temporal bins and minimising the influence of sites with lower chronological resolution (e.g. 300 years) relative to sites with fine scale chronological resolution (e.g. 100 years).

### 3.3 Identifying event horizons, accelerated extractions, and spatiotemporal hotspots

To identify possible thresholds in the adoption of whale resource use (event horizons) and in subsequent accelerated extractions between 1–1900 CE, changes in the number of identified specimens (NISP) will be measured overtime using aoristic sum analysis at three spatial hierarchical levels (country, continent and ocean-basin). Moreover, to identify areas of high concentrations of whale NISP inferred to resemble areas of high resource utilization (herein referred to as spatiotemporal hotspots), NISP counts summed within hex-bins of 5 x 5 degrees longitude and latitude will be used to estimate kernel densities alongside the spatial Getis-Ord Gi* statistics. Gi* statistics use z-scores to measure the degree of spatial clustering with larger z-scores representing regions of increased clustering intensity [183]. Z-scores will be used to identify significant hotspots following [184] with z-scores > 1.65 representing areas with confidence intervals over 90%. Hotspot analysis will be repeated using NISP counts per time interval (< 700 CE; 701–1400 CE; > 1501 CE) to assess changes in hotspots through time and from the global mean. A higher chronological resolution (e.g., counts per century) will be used where feasible.

### 3.4 Identifying cultural and environmental correlates of event horizons, accelerated extractions and spatiotemporal hotspots

To investigate cultural or environmental correlates with identified accelerated extractions or event horizons (if some are determined), cross-correlations with potential explanatory variables, including, proxies of human population size (e.g. cumulative radiocarbon dates) and climatic variables (e.g. sea surface temperature), will be conducted using the R Package *tseries*. In addition, to account for a change in extraction before and after an event (e.g. event horizons), piece-wise linear regressions will be fitted using the same explanatory variables. Moreover, to investigate regions with higher than average whale resource use (spatiotemporal hotspots), distribution occupancy models (presence or absence of whale NISP data within 1 x 1 hexbins) will be created using Bayesian additive regression trees in the R packages dbarts and embarcadero (see [185–187]). Under the assumption that past human societies close to the shore and at locations associated with whale breeding grounds, nursery grounds, or strandings, are more likely to have access to whales as a resource, the following predictor variables will be included: distance from the coast (accepting that this will be an imprecise measure given historical changes in sea level in some regions), latitude, longitude, and altitude. Furthermore, climatic variables that are likely to be correlated with the presence or absence of whale exploitation will be included, namely mean annual sea surface temperatures (SST), annual variability in SST, and isothermality (available from [188,189]). Moreover, as archaeological bone preservation differs based on sediment type, soil pH in H20 and clay content in mass fraction (CLYPPT) will also be included as potential correlates (available from [189]). Sensitivity analysis will be conducted across all data sets to evaluate vis-a-vis data quality scores and qualitative assessments of other potential biases (section 2.5).

## 4 Outputs

Using spatiotemporal mapping of zooarchaeological whale bone data this global systematic review will: (i) document to what taxonomic level faunal material of the great whales is currently published across the globe, and how this varies spatiotemporally; (ii) identify evidence of possible thresholds in the adoption of whale resource use (event horizons), and subsequent accelerated resource utilisation, and potential exploitation of the great whales since 1 CE and prior to the 20th century whaling period (1900 CE onwards); (iii) identify historic hotspots of whale resource use worldwide; (iv) using distribution occupancy modelling identify environmental and climatic correlates of regional whale exploitation; and (v) evaluate to what degree the observed patterns were associated with concurrent socio-economic change. Moreover, using existing biomolecular identification data, combined with knowledge of whale breeding grounds, feeding grounds and common localities of stranding events (through collaborations with zooarchaeologists and cetologists), we will evaluate the spatiotemporal patterns noted above to infer which species may have been utilised a given location. This dataset will also contribute to an open-access global atlas of historical marine resource utilisation.

## Supporting information

S1 Checklist PRISMA-P (Preferred Reporting Items for Systematic review and Meta-Analysis Protocols) checklist. (PDF)

S1 Table Summary of non-english search terms and the number of search results identified using google scholar. (PDF)

S2 Table Metadata of data columns extracted from studies. (PDF)
