# Supplementary material for: Archaeological evidence of resource utilisation of the great whales over the past two millennia: A systematic review protocol
Source: PLoS One. 2023 Dec 14;18(12):e0295604. doi: 10.1371/journal.pone.0295604 (PMC10721060; doi:10.1371/journal.pone.0295604)
Supplement: S1 Table — (PDF) [file pone.0295604.s002.pdf]

Supplementary Material for Manuscript: Archaeological evidence of resource utilisation of the great whales over the past two millennia: A systematic review protocol

DANIELLE L. BUSS[1], YOURI VAN DEN HURK[1], MOHSEN FALAHATI-ANBARAN[1], DEIRDRE ELLIOTT[2], SALLY EVANS[3], BRENNA A. FRASIER[4], JACQUELINE A. MULVILLE[5], LISA K. RANKIN[2], HEIDRUN STEBERGLØKKEN[6], PETER WHITRIDGE[2], and JAMES H. BARRETT[1]

SUPPLEMENTARY TABLE 1. - SUMMARY OF NON-ENGLISH SEARCH TERMS AND THE NUMBER OF SEARCH RESULTS IDENTIFIED USING GOOGLE SCHOLAR

Search Term 1	Search Term 2	Language	No. speakers in year 2020 fromhttps://www.visualcapitalist.com/100-most-spoken-languages/	Average no. hits - google scholar search	> inclusion threshold
ARCHAEOLOGY OR ARTEFACT OR HISTORY	WHALE	English	> 1 billion	40,800; 13,800;418,000	TRUE
考古学 OR 人工制品 OR 历史	鲸	Mandarin chinese	> 1billion	2080; 3540; 6,690	TRUE
पुरातत्त्व OR शिल्पकृति OR इतिहास	व्हेल	Hindi	~0.6 billion	0; 0; 5	FALSE
Arqueología OR Artefacto OR Historia	Ballena	Spanish	~0.5 billion	10,400; 6,500; 34,400	TRUE
Archéologie OR Artefact OR Histoire	Baleine	French	~0.25 billion	6830; 2480; 24,400	TRUE
علم الآثار قطعة أثرية التاريخ	حوت	Arabic	~274 million	442; 3; 74	FALSE
প্রত্নতত্ত্ব প্রত্নবস্তু ইতিহাস	তিমি	Bengali	~265 million	0; 0; 4	FALSE
Археология;	кит	Russian	~258 million	7,650; 2,220; 19,100	TRUE

артефакт;<br>история					
arqueologia;<br>artefato;<br>história	baleia	Portugese	~234 million	5,220; 4,640; 24,500	TRUE
Arkeologi;<br>artefak; sejarah	paus	Indonesian	~199 million	916; 454; 4	FALSE
آثار قدیمہ<br>نوادرات<br>تاریخ	وہیل	Urdu	~170 million	3; 1; 3	FALSE
Archäologie<br>Artefakt<br>Geschichte	Wal	German	~132 million	5340, 7000, 59,800	TRUE
考古学<br>アーティファクト<br>歴史	鯨	Japanese	~128 million	1490, 5110, 13200	TRUE
akiolojia<br>kazi ya sanaa<br>historia	nyangumi	Swahili	~98 million	2, 32, 22	FALSE
पुरातत्व<br>कलाकृती<br>इतिहास	देवमासा	Marathi	~95 million	0, 0, 0	FALSE
పురావస్తు శాస్త్రం<br>కళాఖండం<br>చరిత్ర	తిమింగలం	Telugu	~93 million	0, 0, 0	FALSE
ਪੁਰਾਤੱਤਵ<br>ਕਲਾਤਮਕ<br>ਇਤਿਹਾਸ	ਵ੍ਹੇਲ	Punjabi	~93 million	0, 0, 0	FALSE
考古學<br>人工製品<br>歷史	鯨	Wu Chinese	~82 million	2080, 1180, 6760	TRUE
தொல்லியல்<br>கலைப்பொருள்	திமிங்கிலம்	Tamil	~81 million	0, 0, 0	FALSE

வரலாறு					
arkeoloji<br>eser<br>Tarih	balina	Turkish	~80 million	771, 3020, 4780	TRUE
고고학<br>인공물<br>역사	고래	Korean	~77 million	555, 118, 7900	TRUE
khảo cổ học<br>sự thật<br>lịch sử	cá voi	Vietnamese	~77 million	14900, 14100, 13000	TRUE
Arkeologi;<br>artefak; sajarah	paus	Javanese	~68 million	916; 454; 11	FALSE
Archeologia<br>Artefatto<br>Storia	Balena	Italian	~68 million	631, 816,14200	TRUE
علم الآثار<br>قطعة أثرية<br>التاريخ	حوت	Egyptian spoken Arabic	~65 million	442; 3; 74	FALSE
ilmin kimiya na<br>kayan tarihi<br>kayan tarihi<br>tarihi	whale	Hausa	~63 million	1, 760, 260	FALSE
โบราณคดี<br>สิ่งประดิษฐ์<br>ประวัติศาสตร์	วาฬ	Thai	~61 million	61, 104, 326	FALSE
પુરાતત્વ<br>કલાકૃતિ<br>ઇતિહાસ	વ્હેલ	Gujarati	~61 million	0, 0, 1	FALSE
ಪುರಾತತ್ತ್ವ ಶಾಸ್ತ್ರ<br>ಕಲಾಕೃತಿ<br>ಇತಿಹಾಸ	ತಿಮಿಂಗಿಲ	Kannada	~56 million	0, 0, 0	FALSE
Archeologie<br>Artefact<br>Geschiedenis	Walvis	Dutch*	~23 million	647, 1030, 1990	TRUE
Arkæologi OR ARTEFAKT OR HISTORIE	Hval	Danish*	~5.5 million	303; 259; 3280	TRUE

Fornfrøð Vørur Søga	Hvalur	Faroese*	~50,000	0, 10, 9	TRUE
Itsarnisarsiorneq Artefakt Oqaluttuaq	Arfeq	Greenlandic*	~50,000	0, 0, 0	FALSE
ARKEOLOGI OR ARTEFAKT OR HISTORIE	Hval	Norwegian*	~5.3 million	248; 259; 3280	TRUE
Fornleifafræði Gripur Sögu	Hval	Icelandic*	~375,000	3, 23, 812	TRUE
Not included	Arfivik, arvik, kigutilissua, tikaagullik, tunnulik, Tikaagulliusaarnaq, qipoqqaq	Inuktitut*	< 50,000	23, 87, 0, 12, 7, 1, 8	TRUE
Not included	Aġviq, Aġviġluaq, Usiŋuatchiaq, iŋutuvak	Iñupiaq*	< 50,000	21, 0, 0, 0	TRUE

*Languages of whaling nations not in top 30 most spoken languages
