# Supplementary material for: Archaeological evidence of resource utilisation of the great whales over the past two millennia: A systematic review protocol
Source: PLoS One. 2023 Dec 14;18(12):e0295604. doi: 10.1371/journal.pone.0295604 (PMC10721060; doi:10.1371/journal.pone.0295604)
Supplement: S2 Table — (PDF) [file pone.0295604.s003.pdf]

# Supplementary Material for Manuscript: Archaeological evidence of resource utilisation of the great whales over the past two millennia: A systematic review protocol

DANIELLE L. BUSS[1], YOURI VAN DEN HURK[1], MOHSEN FALAHATI-ANBARAN[1], DEIRDRE ELLIOTT[2], SALLY EVANS[3], BRENNA A. FRASIER[4], JACQUELINE A. MULVILLE[5], LISA K. RANKIN[2], HEIDRUN STEBERGLØKKEN[6], PETER WHITRIDGE[2], and JAMES H. BARRETT[1]

## SUPPLEMENTARY TABLE 2 - METADATA OF DATA COLUMNS EXTRACTED FROM STUDIES

| Data Heading | Data Type | Description | Essential/Optional |
|---|---|---|---|
| 4Oceans_datetime | Date time stamp | Datetime stamp of when the data were added into the main database (generated automatically) | Essential |
| 4Oceans_ID | Integer | Unique identifier of row (generated automatically) | Essential |
| 4Oceans_Input_by | varchar | The full name of the researcher or volunteer inputting the data | Essential |
| Publication_ID | Integer | Unique identifier for the linked publication (generated automatically) | Essential |
| Publication_Title | varchar | The title of the report, thesis, or manuscript where the NISP data was obtained | Essential |
| Publication_Year | numeric | The year that the report was published or made openly available | Essential |
| Publication_Author(s) | varchar | Authors of publications where data is extracted | Essential |
| Publication_FileName | varchar | The filename - if saved locally in the 4Oceans project | Optional |
| Corresponding_author_name | varchar | The name of the corresponding author (or equivalent) | Optional |
| Corresponding_author_email | varchar | The email of the corresponding author (or equivalent) | Optional |
| Publication_DOI | varchar | The publication DOI (if applicable) | Optional |
| Publication_URL | varchar | The publication URL (if applicable) | Optional |
| Publication_unpublished_citation | varchar | Appropriate citation for unpublished literature that is available in the public domain (discovered using forward or backward chasing of the peer-reviewed literature) | Optional |
| Publication_citation | varchar | Published zooarchaeology reference(s); the preferred style is BMC. Found at: https://www.biomedcentral.com/getpublished/writing-resources/references | Essential |
| Publication_archaeological_reference | varchar | Archaeological source(s) providing chronological and/or contextual data if not provided in the original reference file; the | Optional |

| | | | |
|---|---|---|---|
| | | preferred style is 'BMC'. | |
| Publication_faunal_analyst_name | varchar | The full name(s) of who did the identifications and/or reporting | Optional |
| Publication_faunal_analyst_email | varchar | The email address(es) of the publication analyst (if differing from the corresponding author(s). | Optional |
| Publication_IP_status | varchar | The intellectual property status of the data in the row: published; other public access; author permission; institution permission; under embargo; permission requested; permission unavailable; permission declined; ambiguous; no access | Essential |
| Site_ID | integer | Unique identifier of site (generated automatically) | Essential |
| Site_name | varhcar | The published or accepted name of the archaeological site, in the local language. When no site_name is provided, this will be automatically generated using the first author's last name appended with the publication year and a sequential numeric value for each site related with this publication. | Essential |
| Site_code | varchar | Some heritage agencies (e.g. city archaeological units) assign these; in other cases they are inventions of our own; left blank if irrelevant | Optional |
| Site_latitude | geo | Numeric value between -90.00 and 90.00 representing the latitude of the site. | Essential |
| Site_longitude | geo | Numeric value between -180.00 and 180.00 representing the longitude of the site. | Essential |
| Site_georef | varchar | A text description of how the georeference was discovered; for example, a national archaeological database; or an excavation report map plus Google Earth. If provided by the primary source, enter 'as authors recorded'. | Essential |
| Site_location_QC | Integer | 1. Faunal remains without location data (e.g. museum specimens with unknown provenance) will be classified as spatially uninformative (0).<br>2. Faunal remains associated with a broad spatial scale (e.g. country, province, state) will be classified as data quality (1).<br>3. Faunal remains lacking a site-specific georeference, but that can be located to within 1 degree of latitude and longitude (for example, attributed to a known modern settlement without specific site coordinates), or where specific site coordinates are provided but of a resolution of 1 degree latitude and longitude, will be classified as data quality (2).<br>4. Faunal remains associated with specific site coordinates (or described location that can be used to derive such coordinates) with precision below 1.0 degree latitude and longitude will be classified as data quality (3). | Essential |
| Site_name_contemporaneous | varchar | The modern name of the settlement in which the zooarchaeological data was recorded; often applicable when an assemblage is from a town or large village; leave blank if not known or irrelevant | Optional |
| Site_country | varchar | Self-evident, except that we will sometimes use nations within | Essential |

| | | | |
|---|---|---|---|
| | | political unions (e.g. Scotland within Great Britain) | |
| Site_country_code | varchar | Automatic assignment of global standardized alpha-3 country codes (where applicable) | Essential |
| Site_county_province_state | varchar | The name of the province/county/region/state as locally defined | Optional |
| Site_county_province_state | varchar | Level of provincial data as locally defined (e.g. county, region, state, province) | Optional |
| Site_type | varchar | The type of site that was excavated (e.g. ship wreck, archaeological settlement, rescue excavation). 'Unknown' for entries with no obvious type. | Essential |
| Site_notes | varchar | Open text column for additional important notes on the site | Optional |
| Assemblage_ID | integer | Unique identifier of assemblage (generated automatically) | Essential |
| Assemblage_name | varchar | If sites have multiple assemblages related to different time periods/chronologies/contexts. This field will split the site data into the assemblages as locally defined. If only one site exists, entry will consist of the site name appended by a number (e.g. London_1) | Essential |
| Assemblage_type | varchar | The type of assemblagesite that was excavated (e.g. ship wreck, settlement). 'unknown' for entries with no obvious type. | Essential |
| Assemblage_date | varchar | The best reported archaeological date of the assemblage, in the terminology used locally (e.g. Roman Iron Age; Middle Saxon; 14th century; early 14th century) | Essential |
| Assemblage_Start_date_CE | Numeric | The numerical start date of the assemblage in years CE; N.B. unless an alternative approach is justified, follow this default model for converting early/mid/late terminology: early 14th C = 1300-1350; mid 14th C = 1325-1375; late 14th C = 1350-1400 | Essential |
| Assemblage_End_date_CE | Numeric | The numerical end date of the assemblage in years CE; N.B. unless an alternative approach is justified, follow this default model for converting early/mid/late terminology: early 14th C = 1300-1350; mid 14th C = 1325-1375; late 14th C = 1350-1400 | Essential |
| Assemblage_Chronology | varchar | Will be automatically generated as the following: Start_date_CE - End_date_CE | Essential |
| Assemblage_Chronological_method | varchar | A description of the chronological method that was used to date the assemblage. If unavailable please complete this field with "Indeterminate" | Essential |
| Assemblage_Chronological_method_QC | Integer | Faunal remains are often associated with chronological information gathered from contexts using biogeochemical methods, such as dendrochronology and radiocarbon dating, making it possible to infer likely time periods.<br>1. Assemblages with no reported date will be classified as temporally uninformative (0).<br>2. Assemblages with an estimated date, but when the dating method used is not clearly reported, will be classified as data quality (1). | Essential |

| | | | |
|---|---|---|---|
| | | 3. Sites (and associated assemblages) that were dated using typology, stratigraphy and/or chronometric methods, but without quantified and up-to-date estimates of error, will be classified as data quality (2).<br>4. Sites using chronometric methods that report primary data (e.g. radiocarbon assays that can be recalibrated) will be classified as data quality (3).Where uncalibrated radiocarbon dates are available they will be recalibrated following current best practice in downstream analysis. | |
| Assemblage_Chronology_notes | varchar | Open text field for any other comments related to the chronology of the assemblage or site. If radiocarbon dated, please reported the delta R or any pertinent information for how the calibrated radiocarbon date was calculated. Additionally, please record the instrument that the radiocarbon date was measured with (if available). | Optional |
| Assemblage_4Oceans_chronology | varchar | Automatically generated lumped dating info from chronological information above. | Essential |
| Assemblage_Chronology_radiocarbon_cal_date | Numeric | The calibrate radiocarbon date. Often expressed as years before 1950 (BP). | Optional |
| Assemblage_Chronology_radiocarbon_uncertainity | Numeric | The uncertainty around the calibrated date (reported in Assemblage_Chronology_radiocarbon_cal_date). Often referred to as "Calibrated age range" | Optional |
| Assemblage_Chronology_radiocarbon_uncal_date | Numeric | The raw measurement of the ratio of C-14 to C-12 in the sample relative to the atmosphere. Often expressed as years before 1950 (BP). | Optional |
| Assemblage_Chronology_radiocarbon_labcode | Varchar | Unique identifier of the laboratory that conducted the radiocarbon dating (e.g. TRa) | Optional |
| Assemblage_DataType | varchar | Whether the NISP data is coming from zooarchaeological data, artefacts, both, or other | Essential |
| Assemblage_NISP_Data_Available | boolean | Notes as to whether NISP data is available for a given assemblage and/or site | Essential |
| Assemblage_Total_NISP | Numeric | Sum of all NISP with taxonomic information recorded/reported at the given assemblage (i.e. excluding unidentified) | Optional |
| Assemblage_Total_NISP_Level | varchar | The level that total nisp was recorded. E.g) All taxa grouped; Mammalia only; Cetacea only | Optional |
| Assemblage_Total_NISP_Mammals | Numeric | Sum of all mammalian NISP with taxonomic information recorded/reported at the given assemblage (i.e. excluding unidentified) | Essential |
| Assemblage_Total_Unidentified_Specimens | Numeric | If available, the sum of all specimens recorded for a given assemblage that were not taxonomically identified. | Optional |
| Assemblage_Total_Unidentified_Specimens_Level | varchar | Essential field if Assemblage_Total_Unidentified_Specimens is completed. The level that the total of unidentified specimens was recorded. E.g) All taxa grouped; Mammalia only; Cetacea only; Pinniped only | Optional |
| Assemblage_Total_Specimens | Numeric | Sum of all NISP and unidentified specimens recorded at a given assemblage (Sum of fields Assemblage_Total_NISP and | Optional |

| | | | |
|---|---|---|---|
| | | Assemblage_Total_Unidentified_Specimens) | |
| Assemblage_skeleton | varchar | Comment about whether there was a full or partial skeleton included in NISP data. Possible values - Yes - full, Yes - partial, No, Unknown | Essential |
| Assemblage_sieved | Boolean | Was the archaeological site sieved to identify faunal material. This is unlikely given the nature of whalebones but as we will be recording the total number of unidentified specimens it will be useful to know where smaller taxa (e.g. fishbones) may be resulting in large numbers of unidentified specimens relative to sites where no sieving was performed. | Optional |
| Assemblage_type | varchar | Context; Feature; Phase; Period. Definitions as follows: context – a defined stratigraphic unit relating to a single deposition event with associated chronological information; Feature – a single functional or architectural unit often in the form of a solid feature made up of several contexts; Phase – a logical collection of archaeological contexts (and/or features) recorded during an archaeological excavation with an associated chronological range; Period – either a collection of archaeological phases whereby faunal data has been aggregated among multiple phases, or a series of faunal remains associated with a broad time period with no other contextual information (e.g. Bronze Age). | Essential |
| Assemblage_type_details | varchar | List the reported codes for the periods, phases, features or contexts included within this given assemblage. | Optional |
| Assemblage_timespan | Integer | 1. Assemblages with a chronological range of >500 years will be classified as temporally uninformative (0).<br>2. Assemblages with a chronological range of 301-500 years will be classified as data quality (1).<br>3. Sites with a chronological range of 201-300 years will be classified as data quality (2).<br>4. Sites with a chronological range of ≤200 years will be classified as data quality (3). | Essential |
| Assemblage_notes | varchar | Open text column for additional important notes on the assemblage | Optional |
| Taxon_ID | Integer | Unique identifier of taxon | Essential |
| Taxon_name_publication | varchar | Verbatim name (scientific if both scientific and common name provided) of the taxon as provided by the author of the original publication. | Essential |
| Taxon_Great_Whale | boolean | Whether the verbatim name classifies as one of the great whales (i.e., sperm whale, *Physeter macrocephalus,* Cachalot, Pot-whale, Leviathan, Mysticeti, baleen whale, toothless whale, rorqual, Balaenopteridae, Balaeneidae, Eubalaena, humpback whale, *M.novaeangliae* (and all other species of Mysticeti), large cetacea, unidentified large whale, large whale, Noting the terms whale or cetacea will not be classified as Great_Whale. Alongside the non-english terms for whale presented in Supplementary Table S1. | Essential |
| Taxon_Cetacea | boolean | As above but including all unidentified cetacea and whale of any size. | Essential |
| Taxon_NISP | numeric | Total number of specimens recorded for that given taxon within a given assemblage | Essential |
| Taxon_present | boolean | True of False indicator of whether a taxon was present within the given assemblage. This will always be true if NISP data are provided but may also be true if an object or artefact made out of | Essential |

| | | | |
|---|---|---|---|
| | | whale bone or baleen is referred to in the text described (e.g. whale bone used to construct a house). | |
| Taxon_present_details | varchar | When a taxon is present but not as NISP (e.g. one whale bone used to construct a house; or an artefact made out of whale bone was reported in the text). | Optional |
| Taxon_classification_QC | Integer | 1. Faunal identifications classified as 'Unidentified marine mammal or cetacean' will be classified as taxonomically uninformative (0).<br>2. Faunal identifications classified as 'Unidentified large whale' or equivalent will be classified as data quality (1).<br>3. Faunal identifications of greater taxonomic resolution (e.g. *Mysticeti, Balaenidae, Physeteridae*, *Megaptera novaeangliae*) based on zooarchaeological assessments alone will be classified as data quality (2).<br>4. Faunal identifications to a scientific taxon made using ZooMS, aDNA or specific morphological criteria will be classified as data quality (3). | Essential |
| Taxon_GBIF | numeric | GBIF code for associated taxonomic level reported in taxon_level. | Optional |
| Whaling__Equipment_Presence | boolean | The presence or absence of whaling hunting equipment and/or a record of whale hunting at that location/time period. | Optional |
| Whaling_Equipment_Notes | varchar | A description of the equipment identified and/or the documentation of the hunting. | Optional |
